# Dynamic Dyssynchrony and Impaired Contractile Reserve of the Left Ventricle in Beta-Thalassaemia Major: An Exercise Echocardiographic Study

**DOI:** 10.1371/journal.pone.0045265

**Published:** 2012-09-18

**Authors:** Yiu-fai Cheung, Wei Yu, Shu-na Li, Wendy W. M. Lam, Yuen-chi Ho, Sophia J. Wong, Godfrey C. F. Chan, Shau-yin Ha

**Affiliations:** 1 Division of Paediatric Cardiology, Queen Mary Hospital, The University of Hong Kong, Hong Kong, China; 2 Department of Radiology, Queen Mary Hospital, The University of Hong Kong, Hong Kong, China; 3 Division of Paediatric Haematology and Oncology, Queen Mary Hospital, The University of Hong Kong, Hong Kong, China; Temple University, United States of America

## Abstract

**Background:**

Performance of the left ventricle during exercise stress in thalassaemia patients is uncertain. We aimed to explore the phenomenon of dynamic dyssynchrony and assess contractile reserve in patients with beta-thalassaemia major and determine their relationships with myocardial iron load.

**Methods and Results:**

Thirty-two thalassaemia patients (16 males), aged 26.8±6.9 years, without heart failure and 17 healthy controls were studied. Their left ventricular (LV) volumes, ejection fraction, systolic dyssynchrony index (SDI), and myocardial acceleration during isovolumic LV contraction (IVA) were determined at rest and during submaximal bicycle exercise testing using 3-dimensional and tissue Doppler echocardiography. Myocardial iron load as assessed by T2* cardiac magnetic resonance in patients were further related to indices of LV dyssynchrony and contractile reserve. At rest, patients had significantly greater LV SDI (p<0.001) but similar IVA (p = 0.22) compared with controls. With exercise stress, the prevalence of mechanical dyssynchrony (SDI>4.6%, control+2SD) increased from baseline 25% to 84% in patients. Δ SDI_exercise-baseline_ correlated with exercise-baseline differences in LV ejection fraction (p<0.001) and stroke volume (p = 0.006). Compared with controls, patients had significantly less exercise-induced increase in LV ejection fraction, cardiac index, and IVA (interaction, all p<0.05) and had impaired contractile reserve as reflected by the gentler IVA-heart rate slope (p = 0.018). Cardiac T2* in patients correlated with baseline LV SDI (r = −0.44, p = 0.011) and IVA-heart rate slope (r = 0.36, p = 0.044).

**Conclusions:**

Resting LV dyssynchrony is associated with myocardial iron load. Exercise stress further unveils LV dynamic dyssynchrony and impaired contractile reserve in patients with beta-thalassaemia major.

## Introduction

Iron-induced cardiomyopathy is well documented in patients with beta-thalassaemia major [1]. Early detection of subclinical cardiac dysfunction for timely intervention is hence of paramount importance. In the unmasking of subtle ventricular dysfunction, the role of exercise stress is increasingly acknowledged. In particular, dynamic mechanical dyssynchrony [2] and impaired contractile reserve [3] of the left ventricle during exercise stress have been demonstrated in patients with idiopathic and secondary cardiomyopathies. Nonetheless, studies of left ventricular (LV) function in thalassaemia patients have primarily been performed during resting condition.

In patients with beta-thalassaemia major, substrate for development of LV dynamic dyssynchrony potentially exists. In dilated cardiomyopathy, development of ventricular dyssynchrony has been attributed to progressive myocardial fibrosis [4], [5]. Histological examination of myocardial biopsies from thalassaemia patients has revealed variable degree of fibrosis [6], while up-regulated expression of genes involved in fibrosis has been demonstrated in iron-overloaded cardiomyocytes [7], Evaluation of resting uncoordinated contraction of different LV segments and its exaggeration during exercise stress with possible haemodynamic consequences has been facilitated by the introduction of three-dimensional (3D) echocardiography [8], [9]. Data in thalassaemia patients in this regard are nonetheless lacking.

Assessment of contractile reserve using ejection-phase indices is difficult in the setting of thalassaemia, as these indices are strongly load-dependent. Recently, non-invasive assessment of changes in myocardial acceleration during isovolumic LV contraction (IVA), a relative load-independent index of contractility, with increased heart rate during exercise stress has been shown to be useful in assessing contractile reserve based on the force-frequency relationship [3].

In the present study, we explored the phenomenon of dynamic dyssynchrony and assessed contractile reserve, using respectively exercise three-dimensional and tissue Doppler echocardiography, in patients with beta-thalassaemia major. We further determined in these patients the relationships between myocardial iron load and baseline LV mechanical dyssynchrony and exercise-induced changes in LV dyysynchrony and IVA.

## Methods

### Ethics Statement

The Institutional Review Board of the University of Hong Kong/Hospital Authority Hong Kong West Cluster approved the study and all subjects gave written, informed consent.

### Subjects

Patients with beta-thalassaemia major and without overt congestive heart failure were recruited from the haematology clinic. Healthy non-paid subjects, including siblings and staff volunteers, were recruited as controls. The body weight and height were measured and body surface area was calculated accordingly. The Institutional Ethics Committee approved the study and all subjects gave informed consent.

All subjects had echocardiographic evaluation during rest condition and supine bicycle exercise. In addition, patients underwent T2* cardiac magnetic resonance imaging for determination of myocardial iron load. In patients, cardiovascular assessment was performed within 2 weeks of blood transfusion so as to minimize potential confounding influence of anaemia. Their surface electrocardiograms were assessed for QRS prolongation and evidence of cardiac arrhythmias.

### Tissue-Doppler Imaging

Pulsed tissue Doppler imaging of the apical 4-chamber view was performed with the sample volume positioned at the junction of the LV free wall and mitral annulus. The following parameters were determined: systolic (s) and early (e) and late (a) diastolic annular velocities, e/a ratio, and IVA [3].

### Real-time 3-dimensional Echocardiography

Real-time 3D echocardiographic imaging of the left ventricle was performed from the apical view using a matrix array transducer (Vivid 7 Ultrasound System, General Electric, Vingmed, Horten, Norway). Full-volume acquisition with capturing of 4 adjacent sub-volumes over 4 consecutive cardiac cycles was performed during breathhold.

Offline analysis was performed using commercial 4D analysis software (Tomtec Imaging Systems, Unterschleisheim, Germany). Three slices (2-, 3- and 4-chamber planes) of the left ventricle at end-systole and end-diastole were defined and the endocardial border in each of the slice was traced semi-automatically. A LV cast was created for derivation of LV end-diastolic and end-systolic volumes, based on which the ejection fraction, stroke volume and cardiac output were determined.

The LV cast was then divided pyramidal subvolumes based around a non-fixed central point, and the time-volume data corresponding to each of the 16 standard myocardial segments as defined by the American Society of Echocardiography were obtained. Left mechanical mechanical dyssynchrony was quantified by the systolic dyssynchrony index (SDI), calculated as the SD of time to reach minimum regional volume for each of the segments as a percentage of the cardiac cycle duration [8].

### Exercise Testing

Exercise testing was performed with a supine bicycle according to the McMaster cycle ergometer protocol. The initial workload was 25 W with a 25 W increase in resistance at 2-minute intervals. Three-dimensional echocardiographic data for assessment of ventricular dyssynchrony and LV IVA were collected at baseline and when the heart rate reaches 70% of the age-predicted peak heart rate. Given the well documented impaired exercise capacity of thalassaemia patients with significant iron overload [10], [11], a submaximal exercise testing protocol was adopted.

### Magnetic Resonance Imaging

The T2* scan was performed by a 1.5T Siemens Sonata scanner as described previously [12], [13]. Briefly, a four-element cardiac phased array coil was used to image a single 10 mm mid-ventricular short axis slice at 12 echo times with ECG gating. Double inversion recovery pulses were applied to suppress the blood signal. Data were acquired every other cardiac cycle. For T2 measurement, a region of interest was selected in the LV septum. The mean signal intensity of the region of interest was measured for each of the images, and the data were plotted against echo times for derivation of a decay curve. The mono-exponential decay model and the nonlinear curve fitting algorithm were used to fit the curve to obtain the T2 measurements.

### Statistical Analysis

All data are presented as mean±SD. The LV volumes, stroke volume and cardiac output were indexed by body surface area. The IVA-heart rate slope was calculated by the formula: ΔIVA_exercise-baseline_/Δheart rate_exercise-baseline_. Analysis of variance with two factors was used to determine the effect of group (patients vs controls) or the effect of exercise (baseline vs exercise) or interaction of the two. Comparison of demographic variables and delta changes from baseline to exercise of echocardiographic variables between patients and controls were performed using unpaired Student’s t test. Left ventricular mechanical dyssynchrony was defined as LV SDI exceeding mean+2SD as derived from baseline control data at rest. Multiple linear regression analysis was used to determine significant correlates of IVA-heart rate slope. The intra- and interobserver variabilities of baseline and exercise LV ejection fraction and SDI were reported as coefficients of variation, calculated by dividing the SD of differences between measurements by the mean and expressed as a percentage. One of the coauthors (WY) performed repeated measurements in the same offline analysis session to assess intraobserver reproducibility, while two readers (WY and SJW) performed blinded repeated measurements to determine interobserver variability. Within group analyses using Pearson correlation was performed to assess for associations between the cardiac T2* findings and baseline LV dyssynchrony, ejection fraction, changes in dyssynchrony index during exercise stress, and contractile reserve. A p value <0.05 was considered statistically significant. All statistical analyses were performed using SPSS version 11.5 (SPSS, Inc., Chicago, Illinois).

## Results

### Subjects

Thirty-two patients (16 males) aged 26.8±6.9 years were studied. The types of chelation therapy at the time of study included deferiprone with deferoxamine in 20 patients, deferoxamine in 5 patients, deferasirox in 5 patients, and deferiprone in 2 patients. A total of 17 healthy subjects (7 males), aged 25.3±4.8 years (p = 0.43), were recruited as controls. Compared with controls, patients were lighter (p = 0.045) and having a smaller body surface area (p = 0.013) (Table 1). All of the subjects were non-smokers. None of the patients had prolonged QRS duration and electrocardiographic evidence of cardiac arrhythmias or bundle branch block.

**Table 1:** Characteristics of subjects.

	Patients	Controls	p
	(n = 32)	(n = 17)	
Age	26.8±6.9	25.3±4.8	0.43
Sex (male:female)	16∶16	7∶10	0.76
Body weight (kg)	51.9±9.9	58.3±11.2	0.045*
Body height (cm)	159.4±8.7	167.3±7.9	0.003*
Body surface area (m^2^)	1.51±0.17	1.65±0.17	0.013*
Co-morbidities	Hypogonadism (n = 20)	–	
	Hepatitis C infection (n = 9)		
	Diabetes mellitus (n = 6)		
	Hypothyroidism (n = 6)		
	Hypoparathyroidism(n = 1)		

* Statistically significant.

### Resting and Exercise Echocardiographic Findings

Table 2 summarizes the baseline and exercise echocardiographic findings in patients and controls. The coefficients of variation for intra- and inter-observer measurements of LV ejection fraction were respectively 2.5% and 3.5% at rest, and 2.8% and 5.8% when target heart rate was reached during exercise. All of the patients and controls attained the target of 70% of the age-predicted peak heart rate without the need to terminate prematurely the submaximal exercise testing.

**Table 2:** Comparisons of echocardiographic parameters between patients and controls at baseline and during supine bicycle exercise.

	Baseline		Exercise		p		
	Patients	Controls	Patients	Controls	Group	Exercise	Interaction
	(n = 32)	(n = 17)	(n = 32)	(n = 17)	factor	factor	
**Mitral annular tissue Doppler parameters**							
s (cm/s)	6.2±2.1	7.9±1.5	10.1±2.8	11.0±2.0	0.006*	<0.001*	0.38
e (cm/s)	9.9±3.2	10.3±2.0	13.2±3.2	13.1±1.9	0.87	<0.001*	0.72
a (cm/s)	4.0±1.9	4.2±1.5	7.8±4.0	7.4±3.7	0.87	<0.001*	0.67
e/a	2.8±1.0	2.8±1.3	2.2±1.3	2.3±1.3	0.91	0.033*	1.0
IVA (cm/s^2^)	1.1±0.4	1.3±0.5	2.1±0.6	2.9±1.0	<0.001*	<0.001*	0.019*
**LV 3D echocardiographic parameters**							
Indexed EDV (ml/m^2^)	70.2±14.7	66.6±9.6	61.5±10.0	67.0±13	0.73	0.11	0.08
Indexed ESV (ml/m^2^)	31.8±8.0	28.5±4.1	26.9±4.9	24.9±5.0	0.037*	0.001*	0.63
EF (%)	54.8±5.3	57.1±3.8	56.1±4.5	62.6±4.7	<0.001*	0.002*	0.042*
Indexed SV (ml/m^2^)	38.5±8.6	38.1±6.7	34.6±6.7	42.1±9.6	0.036*	0.98	0.021*
Cardiac index (l/min/m^2^)	3.1±0.9	2.8±0.7	4.4±1.0	5.4±1.5	0.098	<0.001*	0.007*
**Heart rate (/min)**	79±13	74±13	128±15	129±16	0.47	<0.001*	0.32

* Statistically significant.

a, mitral annular late diastolic myocardial tissue velocity; e, mitral annual early diastolic myocardial tissue velocity; EDV, end-diastolic volume; ESV, end-systolic volume; IVA, isovolumic acceleration; s, mitral annular systolic myocardial tissue velocity; SV, stroke volume.

At baseline, patients had lower mitral annular s velocity and LV IVA compared with controls. On the other hand, the two groups had similar mitral annular e and a velocities, and e/a ratio. The LV ejection fraction was lower and indexed LV end-systolic volume was greater in patients than controls, while the indexed LV end-diastolic volume and cardiac index were similar between the two groups.

The supine bicycle exercise testing parameters are summarized in table 3. During supine bicycle exercise, all of the mitral annular tissue Doppler velocities, LV IVA, and LV ejection fraction showed a significant increase in patients and controls (table 2). Significant interactions were shown in terms of LV IVA, ejection fraction, indexed stroke volume, and cardiac index, demonstrating a significantly smaller magnitude of increase in these parameters in thalassaemia patients during exercise. Indeed, as also shown in table 4, the absolute changes from baseline to exercise of LV IVA, ejection fraction, indexed stroke volume, and cardiac index were significantly less in patients than controls.

**Table 3:** Exercise testing parameters.

	Patients	Controls	p
	(n = 32)	(n = 17)	
Baseline heart rate(/min)	79±13	74±13	0.18
Peak heart rate (/min)	128±15	129±16	0.86
Baseline SBP (mmHg)	114±9	122±11	0.01*
Baseline DBP (mmHg)	70±7	73±10	0.18
Peak SBP (mmHg)	146±14	159±21	0.052
Peak DBP (mmHg)	77±9	81±11	0.22
Duration of exerciseto achieve targetheart rate (minutes)	11±3	20±4	<0.001*

* Statistically significant.

DBP, diastolic blood pressure, SBP, systolic blood pressure.

**Table 4:** Differences (Δ) between exercise and baseline echocardiographic parameters.

	Patients	Controls	p
	(n = 32)	(n = 17)	
**Mitral annular tissue Doppler parameters**			
Δs (cm/s)	4.0±2.3	3.1±2.6	0.26
Δe (cm/s)	3.3±3.7	2.8±2.2	0.66
Δa (cm/s)	3.8±3.5	3.3±3.5	0.61
Δe/a	−0.0±1.3	−0.6±1.7	1.0
ΔIVA (cm/s^2^)	0.9±0.5	1.6±0.8	<0.001*
**LV 3D echocardiographic parameters**			
Δindexed EDV (ml/m^2^)	−8.7±10.7	0.4±5.3	0.002*
Δindexed ESV (ml/m^2^)	−4.8±7.5	−3.6±3.7	0.52
ΔEF (%)	1.2±7.1	5.5±5.0	0.032*
Δindexed SV (ml/m^2^)	−3.9±6.7	4.0±5.1	<0.001*
Δcardiac index (l/min/m^2^)	1.4±1.0	2.6±1.1	<0.001*

* Statistically significant.

Abbreviations as in table 2.

### Ventricular Mechanical Dyssynchrony

For reproducibility of LV SDI measurements, the coefficients of variation for intra- and inter-observer measurements were respectively 7.7% and 9.7% at rest, and 11.7% and 11.0% when target heart rate was reached during exercise.

**Figure 1:**
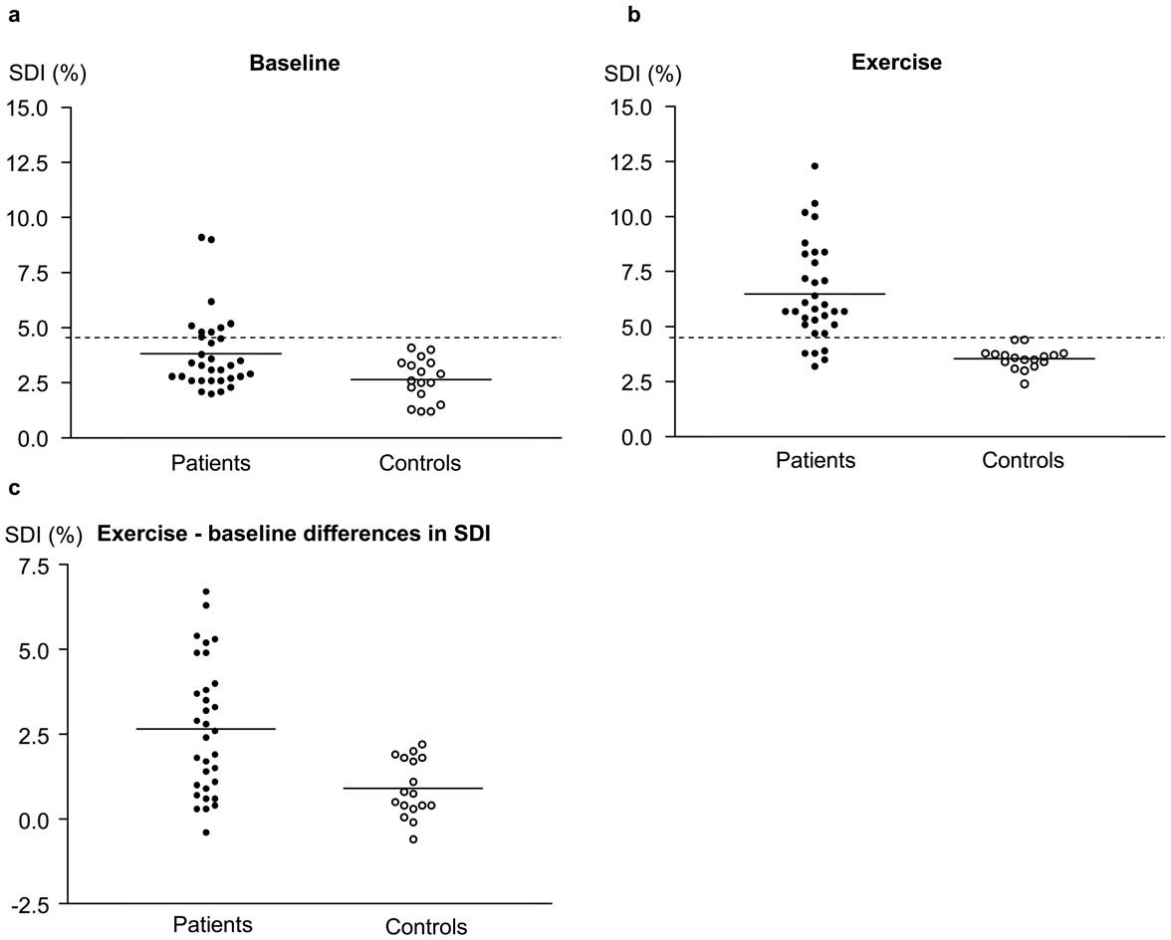
Scatter plots showing (a) baseline left ventricular systolic dyssynchrony index (SDI) at rest, (b) SDI during exercise when target heart rate was reached, and (c) exercise-baseline differences in SDI in patients and controls. Solid horizontal lines represent the mean, while the dotted line represents the upper limit of normal LV SDI.

The resting LV SDI was significantly greater in patients than controls (3.8±1.7% vs 2.6±1.0%, p<0.001) (Fig. 1a). Based on control data, global LV mechanical dyssynchrony was defined as SDI >4.6%. At resting condition, the prevalence of LV mechanical dyssynchrony in patients at rest was 25% (8/32).

In patients, the cardiac T2* finding correlated negatively with baseline LV SDI (r = −0.44, p = 0.011) and positively with LV ejection fraction (r = 0.43, p = 0.014). Using 20 ms as a cutoff for significant myocardial iron overload [14], 12 patients with T2* finding <20 ms had significantly greater resting SDI than 20 with T2* above 20 ms (4.8±2.3% vs 3.2±1.0%, p = 0.01).

**Figure 2:**
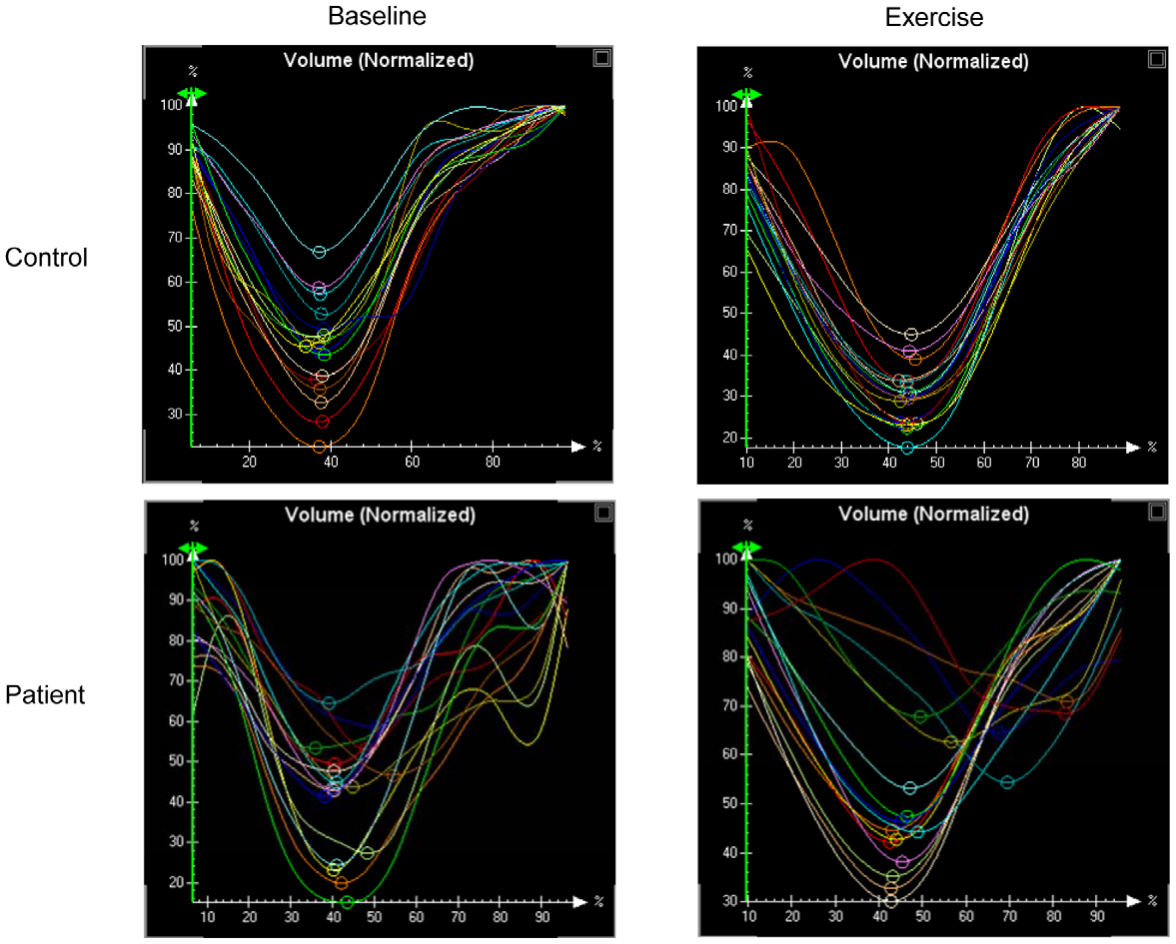
Regional volume curves, normalized to individual maximum, of the 16 LV segments over a cardiac cycle. The upper panels show synchronous contraction of the 16 left ventricular segments in a control at rest and during exercise, while the lower panels demonstrate dyssynchronous contraction at rest and its exaggeration during exercise in a thalassaemia patient.

During exercise, the LV SDI remained significantly greater in patients than controls (6.5±2.2% vs 3.5±0.5%, p<0.001). Analysis of variance similarly revealed significant differences between groups (group factor, p<0.001) and between exercise and baseline (exercise factor, p<0.001) in terms of LV SDI. The prevalence of LV dyssynchrony in patients increased to 84% (27/32) during exercise when the target heart rate was achieved (Fig. 1b). Figure 2 shows examples of changes in time-segmental volume curves of the 16 LV segments throughout the cardiac cycle at baseline and during supine bicycle exercise testing in a control subject and a patient.

**Figure 3:**
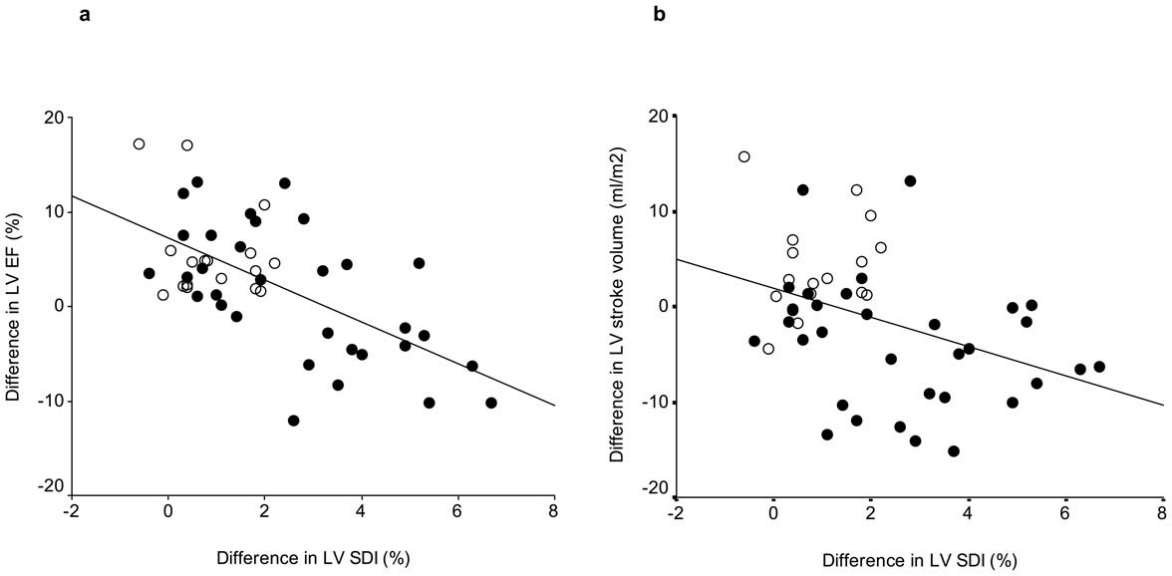
Scatter plot showing a negative correlation between exercise-baseline differences in left ventricular (LV) systolic dyssynchrony index (SDI) and exercise-baseline differences in (a) LV ejection fraction (EF) and (b) indexed LV stroke volume (closed circles: patients, open circles: controls).

Dynamic dyssynchrony was quantified by the difference between exercise and baseline SDI (Δ SDI). Compared with controls, patients had significantly greater Δ SDI (2.6±1.9% vs 0.9±0.8%, p = 0.001; interaction, p = 0.017) (Fig. 1c). ΔSDI correlated negatively with exercise-baseline differences in LV ejection fraction (r = −0.56, p<0.001) and indexed stroke volume (r = −0.39, p = 0.006) (Fig. 3). In patients, ΔSDI correlated positively with T2* (r = 0.48, p = 0.005), although this finding was probably related to worse resting LV dyssynchrony in iron loaded myocardium as shown above, and the tendency for patients with worse baseline LV SDI to have smaller ΔSDI during exercise (r = −0.27, p = 0.13).

### Contractile Reserve

**Figure 4:**
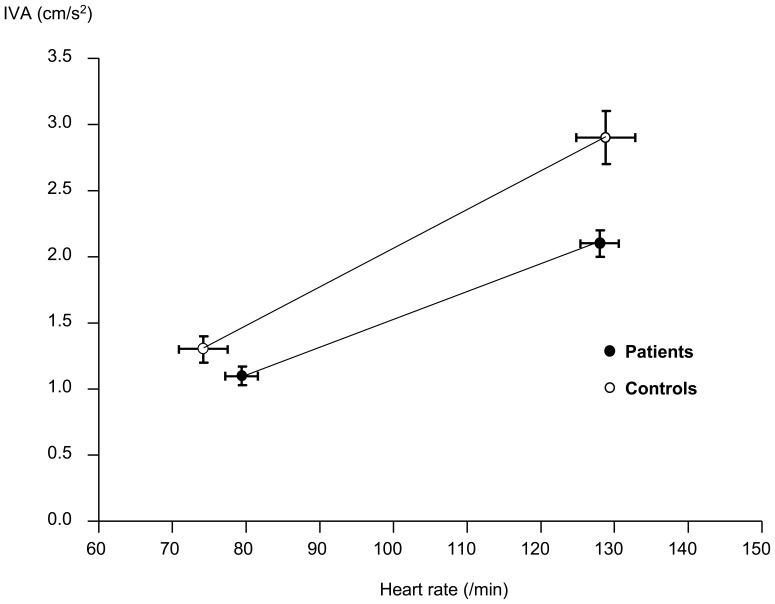
Line plot showing impaired contractile reserve in patients as reflected by the smaller increment of left ventricular myocardial isovolumic acceleration (IVA) with escalating heart rate during exercise in patients than controls. The error bars represent standard errors of means.

The LV IVA, while similar between the two groups at baseline (p = 0.22), was significantly lower in patients during exercise (p = 0.001) (Table 2). Furthermore, the increase in IVA with heart rate was significantly impaired in patients compared with controls (0.022±0.011 cm/s•min vs 0.032±0.018 cm/s•min, p = 0.018) (Fig. 4). In patients, T2* correlated positively with the IVA-heart rate slope (r = 0.36, p = 0.044).

Independent correlates of IVA-heart rate slope in patients as identified from multivariate regression analysis were T2* (beta = 0.37, p = 0.023) and body weight (beta = 0.41, p = 0.014) after adjustment for age, gender, body height, and baseline heart rate, IVA, LV ejection fraction, and exercise-baseline differences in LV ejection fraction.

## Discussion

The novel findings of this study are that in patients with beta-thalassaemia major 1) resting LV mechanical dyssynchrony occurs with a prevalence of 25%, 2) dynamic dyssynchrony and impaired contractile reserve of the left ventricle are evident at even submaximal exercise stress, and 3) baseline LV SDI and contractile reserve during stress correlate with myocardial iron load. To our knowledge, this is first study to assess dynamic changes in LV mechanical dyssynchrony and cardiac contractile reserve in iron-loaded thalassaemia patients during exercise stress.

Dyssynchronous contraction of LV myocardium is recognized as an important factor that may contribute to global LV systolic dysfunction [15]–[17]. Uncoordinated contraction of different LV segments causes ineffective ventricular pressure generation and inefficient transformation of fibre shortening into cardiac output [18]. Three-dimensional echocardiography has been shown to be useful in quantifying LV dyssynchrony in adults with left bundle branch block [19] and those with heart failure undergoing biventricular pacing [20]. Using this technique, we provided the first evidence of not only LV mechanical dyssynchrony at rest but also its unmasking and exaggeration at submaximal exercise testing. The level of reproducibility of 3D echocardiographic index of dyssynchrony at rest and exercise in the present study compares favourably with that reported using tissue Doppler evaluation of 12 LV segments [21].

Dynamic dyssynchrony has been demonstrated in hypertensive heart failure patients with normal ejection [22] and heart failure patients with impaired LV systolic function secondary to coronary heart disease and dilated cardiomyopathy [9], [21], [23] Importantly, dynamic ventricular dyssynchrony has been associated with impairment of increase in LV stroke volume during exercise [2], [23]. Our finding of significant negative correlations between ΔSDI and changes in LV ejection fraction and stroke volume during exercise corroborate the previous findings.

Whether ventricular dyssynchrony is a marker of heart failure or that it contributes to the pathophysiology of heart failure has been a matter of debate. Nonetheless, our finding of ventricular dyssynchrony even in patients without clinical cardiac failure, the reported relationship between ΔSDI and changes in LV output during exercise in this and previous studies [2], [23], the regional molecular changes shown in dyssynchronous failing heart [16], and the demonstration of ventricular dyssynchrony being an independent mechanism of decline in systolic function in hypertensive patients with LV hypertrophy [17], [24] support the contention that dyssynchrony contributes to LV systolic function.

The mechanism of LV dyssynchrony and its exaggeration during exercise stress in thalassaemia patients remains speculative. The possible contribution of progressive patchy fibrosis with evidence from histological examination [6] and in vitro experiments [7] has been alluded to earlier. Indeed, patchy fibrosis has been recently demonstrated in vivo by delayed enhancement cardiovascular magnetic resonance in 24% of the 115 young adult thalassaemia patients studied [24]. Although left bundle branch block has been reported in thalassaemia patients with overt heart failure [25], this is an unlikely explanation in our asymptomatic patients who did not have this electrocardiographic abnormality.

The significant relationship between cardiac T2* measurement and LV SDI at rest suggests that myocardial iron load probably plays an important role in its pathogenesis. Ferrous iron interacts with ryanodine-sensitive calcium channels responsible for the activation of myocardial contraction and modulation of calcium uptake in the sarcoplasmic reticulum [26]. Patchy deposition of iron in the myocardium in iron-overloaded conditions [27] may therefore potentially lead to inhomogeneous myocardial contraction. As ferrous iron and calcium ion share similar charge and size, it is possible that iron overloading of the myocardium affects calcium cycling at higher heart rates during exercise and results in dynamic ventricular dyssynchrony, albeit this remains speculative.

The positive force-frequency relationship, which reflects intrinsic myocardial performance, is related to increased and more efficient calcium cycling as heart rate increases [28], [29]. Utilization of the variability of LV IVA with heart rate during exercise has enabled non-invasive evaluation of intrinsic myocardial performance and contractile reserve [3], [30], [31]. The relative load-independent IVA offers important advantage in evaluating LV function in our patients as their anaemic state may confound assessment using ejection phase indices especially during exercise stress. Our finding of suboptimal increase in LV IVA with escalating heart rate in patients compared with controls suggests impairment of LV contractile reserve, a phenomenon observed similarly in adolescents after anthracyline therapy [3] and paediatric and adult patients with Shwachman–Diamond syndrome [30]. The independent relationship between T2* and LV IVA-heart rate slope in our patients provides further evidence of potential pathogenetic role of myocardial iron.

Our findings of resting and dynamic mechanical dyssynchrony and impaired contractile reserve of the left ventricle and their relationships with myocardial iron load in thalassaemia patients have important clinical implications. Intensification of iron chelation therapy may improve not only resting LV systolic function [32], but potentially also reduce resting and dynamic dyssynchrony and improve contractile reserve. Afterload reduction with angiotensin-converting enzyme inhibitor may improve LV function in thalassaemia patients not only by optimizing ventriculo-arterial interaction [33], but perhaps also through diminishing LV dyssynchrony by reducing wall stress [34] and cardiac fibrosis [35]. Cardiac resynchronization therapy (CRT) is a promising therapy in patients with advanced heart failure [36]. Patients with doxorubicin-induced cardiomyopathy, in whom we have documented evidence of LV mechanical dyssynchrony [37], has been shown to benefit from CRT [38]. Hence, there exists a potential role of CRT in thalassaemia patients with advanced heart failure and documented LV dyssynchrony, especially with the recent availability of cardiac magnetic resonance compatible pacemakers [1], [39].

The cross-sectional design of the present study has its inherent limitations. While the presence of LV dyssynchrony in adults with heart failure has been demonstrated to have prognostic implications in terms of risk of cardiac events and mortality [40], [41] its prognostic value in thalassaemia patients remains to be defined. The relatively small number of patients receiving different types of chelation therapy limits the statistical power of subgroup analyses. Furthermore, reversibility of LV dyssynchrony and impaired contractile reserve with intensification of iron chelation cannot be addressed in this study. We did not include thalassaemia patients with symptomatic heart failure, given the concern of LV dyssynchrony being a marker or a fundamental phenomenon of heart failure [42]. As LV dyssynchrony exists even in our asymptomatic patients, it is perhaps reasonable to speculate that it would probably worsen with onset of congestive heart failure.

In conclusion, we have provided novel data that suggest resting and dynamic mechanical dyssynchrony and impaired contractile reserve of the left ventricle in patients with beta-thalassaemia major. These findings may offer new perspectives for the understanding and management of iron-induced cardiomyopathy.
